# Timing of antiretroviral therapy for HIV-infected patients with cytomegalovirus retinitis: study protocol of a multi-center prospective randomized controlled trial

**DOI:** 10.1186/s13063-021-05159-y

**Published:** 2021-03-18

**Authors:** Xiao-Qing He, Yin-Qiu Huang, Yan-Ming Zeng, Yuan-Yuan Qin, Sheng-Quan Tang, Xiao-Lei Xu, Vijay Harypursat, Yan-Qiu Lu, Min Liu, Jing Yuan, Yao-Kai Chen

**Affiliations:** 1 Division of Infectious Diseases, Chongqing Public Health Medical Center, 109 Baoyu Road, Shapingba District, Chongqing, 400036 China; 2 National Key Laboratory for Infectious Diseases Prevention and Treatment with Traditional Chinese Medicine, Chongqing Public Health Medical Center, Chongqing, 400036 China

**Keywords:** Cytomegalovirus retinitis, Acquired immunodeficiency syndrome, Timing of ART initiation

## Abstract

**Background:**

Cytomegalovirus retinitis (CMVR) is an important opportunistic infection (OI) occurring mainly in patients with acquired immunodeficiency syndrome (AIDS) and has the potential to cause severe visual impairment and blindness among AIDS patients. Subsequent to the adoption and implementation of widespread antiretroviral therapy (ART), the prognosis of AIDS-associated CMVR has been substantially improved. Nevertheless, the equivocal clinical evidence as regards the optimal timing for ART initiation in patients with an established CMVR diagnosis is required. We therefore designed the present study in order to investigate the optimal timing for ART initiation in AIDS/CMVR patients.

**Methods:**

This will be a prospective, randomized controlled trial to be performed at 17 hospitals in mainland China. A total of 300 participants with CMVR will be randomly assigned to an early ART initiation group (ART initiation within 2 weeks after anti-CMV therapy), or a deferred ART initiation group (initiation of ART more than 2 weeks after anti-CMV therapy) at a 1:1 ratio. All participants will receive 48 weeks of follow-up after anti-CMV therapy initiation. Our primary outcome will be the incidence of visual loss (to a visual acuity worse than 20/40 or 20/200) in the two groups during the 48-week follow-up period. Secondary outcomes will include changes in HIV virological suppression and serum CD4^+^ T-cell counts, the incidence of mortality, retinitis progression (movement of the peripheral border of a CMV lesion ≥ ½ disc diameter, or occurrence of a new CMV lesion), retinal detachment, immune recovery uveitis (IRU), and other OIs and adverse events between the two study groups during the 48 weeks of follow-up.

**Discussion:**

The study aims to investigate the optimal timing for ART initiation in AIDS/CMVR patients. We hope to be able to extract robust clinical evidence for use in optimal AIDS/CMVR management should our trial be successful.

**Trial registration:**

This research was registered as one of the twelve clinical trials under the name of a general project “A study for precision diagnosing and treatment strategies in difficult-to-treat AIDS cases and HIV-infected patients with highly fatal or highly disabling opportunistic infections”, ChiCTR1900021195. Registered on 1 February 2019, http://www.chictr.org.cn/showproj.aspx?proj=35362.

## Background

Cytomegalovirus (CMV) is a double-stranded DNA virus in the herpesvirus family of viruses, and CMV infection is highly prevalent in humans, especially in HIV-infected populations. HIV and CMV share similar modes of transmission, and in HIV-infected patients with profound immunosuppression (CD4^+^ T-cell counts < 50 cells/μl), CMV may cause disseminated or localized end-organ disease [1–3]. Among HIV-infected patients with CMV disease, cytomegalovirus retinitis (CMVR) accounts for approximately 85% of cases, and CMV-related gastrointestinal tract disease accounts for 10% of cases, followed by CMV neurological disorders, CMV pneumonia, CMV hepatitis, and CMV adrenolytic [4].

The rate of bilateral blindness (visual acuity < 20/200 in both eyes) resulting from CMVR has decreased from 14.8/100 person-year (PY) prior to the advent of modern antiretroviral therapy (ART) to 0.4/100 PY in the modern ART era [5–7]. However, even in the present era of widespread ART, CMVR continues to cause blindness among patients with AIDS in low and middle income countries, where approximately 30% of patients may experience blindness in one or both eyes due to CMVR [8].

Although ART plays a vital role in the management of AIDS patients with CMVR (AIDS/CMVR), the timing of ART initiation in AIDS/CMVR patients who have started anti-CMV therapy remains unresolved. Domestic and international guidelines recommend initiating ART within 2 weeks of starting anti-CMV therapy in AIDS/CMVR patients [9, 10]. These recommendations, however, are based on expert opinions and are not based on empirical evidence extracted from clinical trials. In addition, the results of one retrospective study indicate that early ART initiation resulted in a higher incidence of immune recovery uveitis (IRU), which may cause visual loss, visual impairment, and other oculo-visual complications [11]. Thus, unequivocal clinical evidence for the timing of ART initiation in AIDS/CMVR patients is urgently required. In the present study, we have designed a prospective, open-labeled, randomized, controlled trial in order to investigate the optimal timing for ART initiation in the AIDS/CMVR population.

## Methods/design

### Study design and setting

This study is a prospective, open-labeled, randomized controlled trial (RCT) that will performed at 17 hospitals in 12 provinces in mainland China: Chongqing Public Health Medical Center, Beijing You’an Hospital of Capital Medical University, the Forth Affiliated Hospital of Harbin Medical University, the Second People’s Hospital of Tianjin, the First Hospital of Changsha, the Eighth People’s Hospital of Guangzhou, Liuzhou General Hospital, the Third People’s Hospital of Guilin, the Third People’s Hospital of Shenzhen, Guiyang Public Health Clinical Center, Public Health Clinical Center of Chengdu, the Third People’s Hospital of Kunming, Yunnan Provincial Infectious Disease Hospital, the Fourth People’s Hospital of Nanning, Guangxi Longtan Hospital, the First Affiliated Hospital of Zhejiang University, and Xixi Hospital of Hangzhou.

**Fig. 1:**
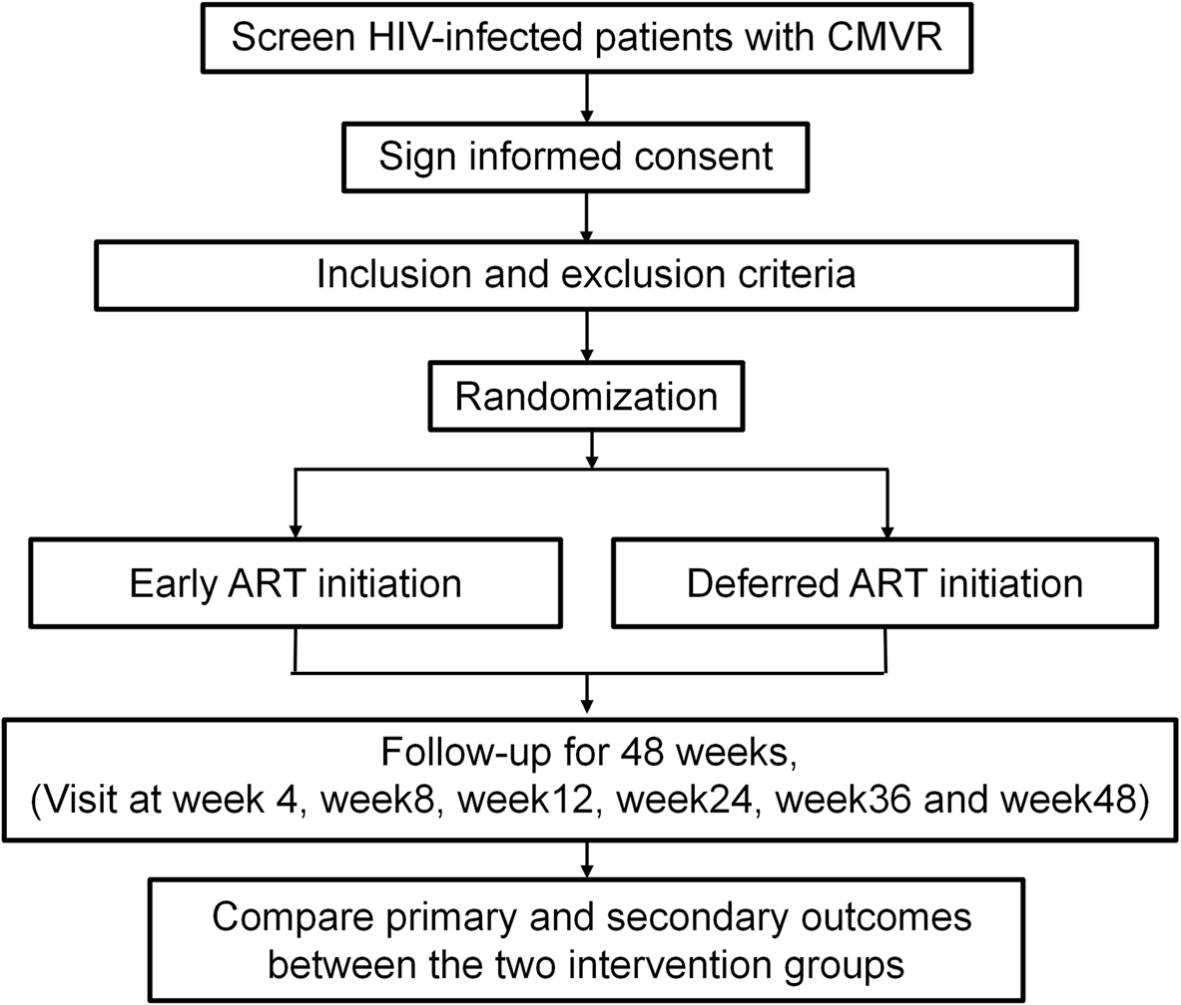
Flow chart of study design

A total of 300 AIDS/CMVR patients will be recruited for this prospective trial. Participants will be randomized into two groups at a 1:1 ratio: an early ART initiation group (initiation of ART within 2 weeks after anti-CMV therapy) and the deferred ART group (initiation of ART more than 2 weeks after anti-CMV therapy). Enrolled subjects will be assessed during a 48-week follow-up period at day 0, week 4, week 8, week 12, week 24, week 36, and week 48. Aqueous or vitreous humor fluid, blood, and urine samples will be collected for laboratory testing, including for hematological analysis, liver and kidney function tests, blood/ aqueous and vitreous humor CMV DNA testing, lymphocyte subset, and HIV-1 RNA viral load test. An experienced ophthalmologist will regularly examine subjects and record individual retinal changes. All blood samples will be analyzed once and then destroyed; specimens will not be stored for future use. Table 1 represents a complete schedule of study activities during the 48-week follow-up period. Schematic outlines of this study design and procedures are shown in Fig. 1. This protocol was approved by the Ethics Committee of Chongqing Public Health Medical Center (2019-003-02-KY) and has been registered as one of the twelve trials under the name of a general project at the Chinese clinical trials registry, the trial registration number being ChiCTR1900021195.

**Table 1:** Study enrollment and follow-up schedule, detailing specific interventions and assessments

Time window	Visit 1	Visit 2	Visit 3	Visit 4	Visit 5	Visit 6	Visit 7	Visit 8
	Screen − 28 to 0 days	Baseline 0 day	4 weeks±14 days	8 weeks±14 days	12 weeks±14 days	24 weeks±14 days	36 weeks±14 days	48 weeks±14 days
Sign informed consent	X							
Inclusion and exclusion criteria	X							
Demography	X							
Signs and symptoms	X		X	X	X	X	X	X
HIV screening	X							
HIV confirmation	X							
Hematological analysis	X		X	X	X	X	X	X
Urinalysis	X							
Clinical chemistry studies	X		X	X	X	X	X	X
Blood CMV antibodies IgG/IgM	X					X		X
Blood/aqueous or vitreous CMV DNA	X		X		X	X	X	X
Urine pregnancy test	X							
Lymphocyte subset	X		X	X	X	X		X
Quantitative plasma HIV-1 RNA	X					X		X
Electrocardiogram	X							
Ophthalmological examination	X		X		X	X	X	X
IRU			X	X	X	X	X	X
Therapeutic regimens (anti-CMV and ART)	X	X	X	X	X	X	X	X
Concomitant medications		X	X	X	X	X	X	X
Adverse events		X	X	X	X	X	X	X

### Objective

The study aims to investigate the optimal timing for ART initiation in AIDS/CMVR patients.

### Diagnostic criteria

According to the 2019 DHHS Guidelines for the Prevention and Treatment of Opportunistic Infections in HIV-Infected Adults and Adolescents [10], and the 2018 Chinese Guidelines for Diagnosis and Treatment of HIV/AIDS [9], the diagnostic criteria for HIV infection and AIDS will have to be met.

The diagnosis of CMVR will have to meet the following criteria:

1. Characteristic retinal changes observed at baseline through a dilated pupil during an ophthalmoscopic examination performed by two experienced ophthalmologists.
2. With or without visual symptoms such as floaters, scotomata, blurred vision, decreased visual acuity, loss of peripheral or central vision.
3. In rare cases, diagnosis of CMVR may be difficult, and reverse-transcriptase polymerase chain reaction (PCR) of aqueous humor or vitreous humor specimens for CMV testing may be useful for establishing the diagnosis.

### Inclusion criteria

The study will include HIV-infected patients who are ART-naïve and 18 years or older and have been diagnosed with CMVR. Prospective participants must be willing to complete and sign informed consent documentation. All of the above criteria will have to be met before inclusion in the study.

### Exclusion criteria

Participants will be excluded from this study if (1) they are intolerant or have allergies to any therapeutic drugs; (2) they have laboratory anomalies viz. hemoglobin (Hb) < 60 g/L, white blood cell count (WBC) < 1.0 × 10^9^/L, neutrophil count (N) < 0.5 × 10^9^/L, platelet count (PLT) < 50 × 10^9^/L, blood amylase (AMS) > 2 × upper normal limit (UNL), serum creatinine (Scr) > 1.5 × UNL, aspartate aminotransferase (AST), alanine aminotransferase (ALT) or alkaline phosphatase (ALP) > 5 × UNL, total bilirubin (TB) > 2 × UNL, and serum creatine phosphokinase (CK) > 2 × UNL; (3) they have other serious disease that may adversely affect the evaluation of the efficacy of therapeutic drugs and prognosis of patients; (4) they are pregnant or breast-feeding women; and (5) they have mental illness, are intravenous recreational drug users, or are non-Chinese nationals.

### Recruitment

Our study will include participants from 17 different hospitals in mainland China, and investigators will strive to ensure that the proposed sample size is attained and maintained. Attending doctors will evaluate and select patients according to inclusion and exclusion criteria specified in this protocol. Eligible participants will be asked to leave their own contact information and family members’ contact information after they sign informed consent. The study investigators will provide a list with names and contact information of the participants to the nurse. At each follow-up visit, the nurse will remind them. At the same time, the investigator will comprehensively educate participants on the importance of follow-up compliance at baseline, and at each follow-up visit, in order to enhance their motivation to participate.

### Randomization

All eligible participants will be randomly assigned to one of two groups (ART initiation within 2 weeks vs ART initiation later than 2 weeks) at a 1:1 ratio by an online medical research tool called Medical Research Platform (http://www.51yyt.org/FrontPage/Index.aspx). The computer-generated, sequentially numbered randomization list (variable block sizes) will be prepared by the trial statistician and incorporated in the online secure database. Randomization will be performed by the research manager at each treatment center, who will have access to the next subject allocation number, but not the entire subject list. Our study is an open-label design, where participants, investigators, Outcome assessors, data assessors, and data analysts were not masked to treatment allocation. Drug treatment will be dispensed to participants in an open-labeled fashion (neither participants nor investigators will be masked to drug treatment allocation). Baseline will be defined as the date of randomization.

### Withdrawal and endpoints

Patients may refuse to take part in, or withdraw their participation from this study at any time. If participants meet any of the following criteria, they should withdraw from further participation in the study: (1) development of serious adverse events or serious adverse reactions, (2) non-adherence to the research protocol, and (3) non-compliance with the follow-up schedule. Should any one of the following occur, the end point of the study will be reached: (1) completion of the prescribed 48-week follow-up schedule as per the research protocol; (2) discontinuation of ART during the study period; and (3) death.

### Intervention

According to the recommendations of the 2018 Chinese Guidelines for Diagnosis and Treatment of HIV/AIDS [9], all participants will receive systemic anti-CMV therapy comprising ganciclovir (intravenous 5 mg/kg every 12 h for 14–21 days, followed by a maintenance dose of ganciclovir at 5 mg/kg/day), or foscarnet (intravenous 60 mg/kg every 8 h for 14–21 days, followed by a maintenance dose of foscarnet at 90 mg/kg/day) for treatment of CMVR. Systemic anti-CMV therapy may be combined with intraocular administration of 1–4 doses of ganciclovir (2 mg/injection), or foscarnet (2.4 mg/injection) for over a period of 7–10 days, should that be necessary. After completion of anti-CMV therapy, all participants will receive conventional prophylactic antiviral drug therapy until CD4^+^ T-cell counts increase to > 100 cells/μL, and continue prophylactic treatment for 3–6 months.

As for HIV treatment regimens, in accordance with local HIV treatment guidelines, Tenofovir (300 mg/d) + Lamivudine (300 mg/d) + Efavirenz (600 mg/d) is the preferred drug combination for AIDS; however other guideline-prescribed drug regimen combinations are optional.

### Outcomes

As for primary outcomes, we will compare the cumulative incidence of visual loss (to worse than 20/40 and to 20/200 or worse) [11]. in the two study groups at 4 weeks, 8 weeks, 12 weeks, 24 weeks, and 48 weeks of treatment. Visual was assessed by VA in each eye separately using logMAR charts presented with standard illumination. Visual loss was defined as worse than 20/40 and 20/200 or worse [11].

The secondary outcomes included the mean difference in CD4^+^ T-cell counts between the two study groups from baseline to week 48, the virological suppression (HIV RNA <50 copies/mL) at different follow-up points from baseline to week 48, the difference in the incidence rate of IRU during the study period, the occurrence of adverse events over 48 weeks, other opportunistic infections (OIs) during the 48 weeks of follow-up, as well as the incidence of retinitis progression (defined as the movement of a border of a CMV lesion at least ½ standard disc diameter along a front ½ disc diameter in size or the occurrence of a new lesion ¼ disc area in size [11]) and retinal detachment during the study period, and the overall mortality at week 48. The outcome of retinitis progression is determined by the occurrence in either eye.

IRU will be diagnosed according to the following criteria: (1) anti-CMV drug treatment is effective, and symptoms and signs of CMVR have improved or disappeared prior to ART initiation; (2) ART is effective, resulting in a decrease in plasma HIV RNA level >1 log_10_ copies/ml, or an increase in CD4+ count of ≥25 cells/μl after ART initiation; and (3) patients show worsening clinical symptoms (such as floaters and/or vision loss, the latter usually of moderate degree, with visual acuities worse than 20/40 but better than 20/200) [12] or ophthalmoscopic signs of CMVR after ART initiation, and the worsening signs may not be explained by therapeutic drug toxicity or newly acquired disease.

### Data collection and analyses

All data will be recorded on case report forms (CRFs) by appropriately trained nurses. Data will simultaneously be uploaded into our database at Medical Research Platform by two investigators, independently. The participants’ clinical information will be collected from baseline to end of follow-up for this study. The study management structure contain three part be checked to ensure data completeness as much as possible. Data that are significantly abnormal or outside the clinically acceptable range (laboratory items exceeding 20% of the normal value) must be explained by the physician with the necessary explanation. Drop-outs and adverse events will be recorded in time. The study monitor will consult and review the required clinical research data. After reviewing all the study data and confirming that the uploaded data is accurate, the project leader and the statistical analysts will perform a data lock. When any serious adverse event occurs or patients do not wish to further participate in the trial, they will be excluded from the trial. The all outcomes analyses will be conducted using data from the intention-to-treat population (ITT), defined as all patients who undergo randomization regardless of non-compliance with the intervention. If necessary, we also plan to analyze the outcomes using the per-protocol (PP) analysis set, which excludes subjects who do not follow the treatment regimens. Individual data will be gathered and stored electronically in stored on a secure database. Participants will receive an ID when entering the study. All data will be linked together, and identify the person by using ID codes. A list of IDs will be kept strictly confidential, and only members of the trial team can access. Screening, baseline, and follow-up data will be collected.

No matter primary outcomes and secondary outcome measures, we will perform a descriptive statistical analysis of organizational and socio-demographic characteristics at baseline in order to assure comparability between the two study groups. Baseline measurements and changes in outcome variables for each study-arm will be presented as means or median. All variables that differ significantly between groups will be adjusted for baseline. Categorical variables will be recorded as numbers and percentages and will be compared using the chi-squared test or Fisher’s exact test. The time-to-event method, using Cox proportional-hazards models, will be used to compare mortality at week 48. All data will be analyzed by Statistical Package for the Social Sciences (SPSS) software, Version 20.0 (IBM SPSS Inc., Chicago, IL, USA). Statistical significance will be assumed if *p* < 0.05. Outcome data will be analyzed once only. Final analysis will take place 48 weeks after the last patient is randomized.

### Data quality assurance

Data quality will be checked regularly by research assistants and overseen by monitors. All modifications will be marked on the case report forms, and data managers will re-check the data before they are officially logged. The database will be locked after all data have been cleaned. If participants withdraw from the trial during the study period, the reasons will be documented, and the dropout rate and dropout reason will be statistically analyzed.

### Sample size

The underlying assumption was a reduction of the primary event rate from 25 to 10% with at least 85% power and an overall two-side alpha level of 0.05. Two-sided Z test with mixed variance was used. A sample size of 114 patients will be needed per group in an early ART initiation group and a deferred ART initiation group. Meanwhile, considering 20% drop-out rate, we plan to randomize 300 participants and have at least 240 participants for analysis. The sample size calculation was conducted using the PASS software version 15 (NCSS, LLC, AQ5, USA).

### Patient safety

Safety will be monitored for 48 weeks in this study, and this monitoring will include occurrence of adverse events (AEs), serious adverse events (SAEs), discontinuation of treatment due to AEs, deaths, individual changes in the monitoring data from baseline, all indicators of drug toxicity in laboratory tests, and ECG changes. The time of AE occurrence, AE duration, and AE severity (mild, moderate, or severe) will be accurately documented in individual participant CRFs. Any prolonged hospitalization, persistent or significant disability or incapacity, or unexpected medical occurrence that results in death will be reported as an SAE and will be required to be reported to the principal investigator (PI) and the ethics committee within 24 h. The adverse event include (1) grade 3 or 4 adverse events which will be graded using the Division of AIDS (DAIDS) Table for Grading the Severity of Adult and Pediatric Adverse Events (version 2.1) [13]; (2) serious adverse events defined by US Food and Drug Administration [14]; and (3) adverse events related to discontinuation of medication or regimen change.

### Administration of the trial

The study management structure contains three part: the principle investigator (PI), a trial management group, and a data monitoring committee. The PI meets with representatives of the Administration Office for National Science and Technology Major Projects once or twice per year. The PI and trial management group member oversee the evolution of the trial in 17 research centers every month by networking, phone call, email, or on-site monitoring. The trial management group is responsible for conducting the trial and will meet monthly to discuss trial progress. The data monitoring committee will review interim safety data and periodically review the conduct of the study. After the trial is completed, the Administration Office for National Science and Technology Major Projects will evaluate the quality of the trial. There is no Stakeholder and Public Involvement Group in this trial.

## Discussion

Current Chinese and international guidelines recommend that ART initiation should occur within 2 weeks after starting anti-CMV therapy for CMVR in order to negate the potential for emergence of other OIs should ART initiation be delayed [9, 10]. However, one observational study suggests that delaying ART initiation until CMVR is controlled may be beneficial in reducing the likelihood of development or severity of immune reconstitution inflammatory syndrome (IRIS) [11]. Thus, the precise timing of ART initiation after starting CMVR therapy remains unclear. CMV viral replication is usually inhibited within 1 to 2 weeks after starting anti-CMV therapy, and the duration of currently recommended CMVR induction therapy is 14 to 21 days. We therefore chose 2 weeks of CMV treatment as a convenient timeline to compare ART initiation options in a multicenter, open-labeled, RCT in order to investigate the optimal timing of ART initiation for AIDS/CMVR patients.

CMVR accounted for 40% of vision loss of 20/200 or worse in patients with AIDS in the early-ART era (the period between 1987 and 1996) [5–7], and general quality of life is severely compromised by loss of visual acuity. We therefore defined incidence of visual loss as the primary outcome of our study. Mortality, retinitis progression, retinal detachment, IRU, CD4^+^ T-cell counts, HIV viral load, AEs, and other OI incidence are defined as secondary outcomes in our study. We will study the changes in these variables in order to determine the optimal timing for ART initiation in patients with CMVR. We expect that the results of this prospective RCT will be able to produce robust evidence for optimal clinical management of patients with AIDS/CMVR.

We have anticipated the emergence of a few challenges during course of the implementation of this study. The number of newly diagnosed AIDS-associated CMVR cases is decreasing locally as a result of widespread modern highly active ART coverage in China. Consequently, it is possible that we may not achieve our target cohort population during the proposed study timeframe. In addition, the 48-week follow-up period may be challenging for some participants, and the drop-out rate may be higher than we have anticipated. Also, it may not be possible to completely negate the subjective choices of individual physicians in the clinical management of study participants in this multicenter trial. In order to overcome these challenges, and to ensure strict adherence to the study protocol during implementation of the study, we will (1) recruit participants from 17 large designated regional hospitals for HIV care in China in order to increase our likelihood of recruiting the specified number of study subjects; (2) comprehensively educate participants on the importance of follow-up compliance at baseline, and at each follow-up visit, in order to enhance their motivation to participate; (3) provide a superior level of consulting service excellence to participants during the study period, and this will extend to the period after study completion; and (4) collect standardized ocular fundus photographs, and forward these to one centralized assessment center for grading, in order to reduce the possibility of interpretation variance and consistency bias.

## Trial status

Enrollment for this study began in March 2019 and is expected to be completed in December 2020. At present, all involved study sites are actively screening for eligible patients, and enrolment is ongoing (protocol version 5, 25 August 2019).

## Data Availability

Not applicable.

## Abbreviations

AEs: Adverse events
AIDS: Acquired immune deficiency syndrome
ALT: Alanine aminotransferase
ALP: Alkaline phosphatase
AMS: Amylase
ART: Antiretroviral therapy
AST: Aspartate aminotransferase
CK: Creatine phosphokinase
CMV: Cytomegalovirus
CMVR: Cytomegalovirus retinitis
CRFs: Case report forms
Hb: Hemoglobin
HIV: Human immunodeficiency virus
IRU: Immune recovery uveitis
IRIS: Immune reconstitution inflammatory syndrome
N: Neutrophil
OI: Opportunistic infection
PI: Principal investigator
PLT: Platelet
PY: Person-year
RCT: Randomized controlled trial
RNA: Ribonucleic Acid
SAEs: Serious adverse events
Scr: Serum creatinine
SPSS: Statistical Package for the Social Sciences
TB: Total bilirubin
ULN: Upper limit of normal
WBC: White blood cell
